# Advancements in synthetic CT generation from MRI: A review of techniques, and trends in radiation therapy planning

**DOI:** 10.1002/acm2.14499

**Published:** 2024-09-26

**Authors:** Mohamed A. Bahloul, Saima Jabeen, Sara Benoumhani, Habib Abdulmohsen Alsaleh, Zehor Belkhatir, Areej Al‐Wabil

**Affiliations:** ^1^ College of Engineering Alfaisal University Riyadh Saudi Arabia; ^2^ Translational Biomedical Engineering Research Lab, College of Engineering Alfaisal University Riyadh Saudi Arabia; ^3^ AI Research Center, College of Engineering Alfaisal University Riyadh Saudi Arabia; ^4^ King Faisal Specialist Hospital and Research Centre Riyadh Riyadh Saudi Arabia; ^5^ School of Electronics and Computer Science University of Southampton Southampton UK

**Keywords:** artificial intelligence, computed tomography (CT), deep learning, MRI to CT, magnetic resonance imaging (MRI), oncology, radiation therapy planning, synthetic CT generation

## Abstract

**Background:**

Magnetic resonance imaging (MRI) and Computed tomography (CT) are crucial imaging techniques in both diagnostic imaging and radiation therapy. MRI provides excellent soft tissue contrast but lacks the direct electron density data needed to calculate dosage. CT, on the other hand, remains the gold standard due to its accurate electron density information in radiation therapy planning (RTP) but it exposes patients to ionizing radiation. Synthetic CT (sCT) generation from MRI has been a focused study field in the last few years due to cost effectiveness as well as for the objective of minimizing side‐effects of using more than one imaging modality for treatment simulation. It offers significant time and cost efficiencies, bypassing the complexities of co‐registration, and potentially improving treatment accuracy by minimizing registration‐related errors. In an effort to navigate the quickly developing field of precision medicine, this paper investigates recent advancements in sCT generation techniques, particularly those using machine learning (ML) and deep learning (DL). The review highlights the potential of these techniques to improve the efficiency and accuracy of sCT generation for use in RTP by improving patient care and reducing healthcare costs. The intricate web of sCT generation techniques is scrutinized critically, with clinical implications and technical underpinnings for enhanced patient care revealed.

**Purpose:**

This review aims to provide an overview of the most recent advancements in sCT generation from MRI with a particular focus of its use within RTP, emphasizing on techniques, performance evaluation, clinical applications, future research trends and open challenges in the field.

**Methods:**

A thorough search strategy was employed to conduct a systematic literature review across major scientific databases. Focusing on the past decade's advancements, this review critically examines emerging approaches introduced from 2013 to 2023 for generating sCT from MRI, providing a comprehensive analysis of their methodologies, ultimately fostering further advancement in the field. This study highlighted significant contributions, identified challenges, and provided an overview of successes within RTP. Classifying the identified approaches, contrasting their advantages and disadvantages, and identifying broad trends were all part of the review's synthesis process.

**Results:**

The review identifies various sCT generation approaches, consisting atlas‐based, segmentation‐based, multi‐modal fusion, hybrid approaches, ML and DL‐based techniques. These approaches are evaluated for image quality, dosimetric accuracy, and clinical acceptability. They are used for MRI‐only radiation treatment, adaptive radiotherapy, and MR/PET attenuation correction. The review also highlights the diversity of methodologies for sCT generation, each with its own advantages and limitations. Emerging trends incorporate the integration of advanced imaging modalities including various MRI sequences like Dixon sequences, T1‐weighted (T1W), T2‐weighted (T2W), as well as hybrid approaches for enhanced accuracy.

**Conclusions:**

The study examines MRI‐based sCT generation, to minimize negative effects of acquiring both modalities. The study reviews 2013‐2023 studies on MRI to sCT generation methods, aiming to revolutionize RTP by reducing use of ionizing radiation and improving patient outcomes. The review provides insights for researchers and practitioners, emphasizing the need for standardized validation procedures and collaborative efforts to refine methods and address limitations. It anticipates the continued evolution of techniques to improve the precision of sCT in RTP.

## 1 INTRODUCTION

Computed tomography (CT) scans are crucial and it is the main tool in some countries like UK for radiation treatment planning and dosage computation due to their high spatial resolution and electron density information. However, they can be influenced by patient movement, altering anatomy, and misalignment with other imaging modalities, leading to errors in treatment planning and dose estimations. Metal implants or prostheses can cause large abnormalities in CT scans, compromising treatment planning precision. High costs and limited access to CT scanners make it difficult to obtain essential imaging for treatment planning, and patients may require recurrent scans to track therapy success. The treatment process involves a combination of CT and other imaging techniques, ensuring individualized planning due to variations in human anatomy, and using advanced algorithms and motion management strategies for improved precision and safety.^1^ In this line of work, combining magnetic resonance imaging (MRI) and CT is becoming more popular in certain situations.^2^ For many disease sites, radiation therapy uses both CT and MRI for treatment planning. While CT scans offer the electron density measurements required for treatment planning, MRI scans offer a better soft tissue contrast that helps distinguish between soft tissues and malignancies. MRI can significantly reduce contouring variability in treatment planning by improving inter‐observer variability, which refers to differences in how medical professionals outline structures on medical images, and intra‐observer variability, which describes variations in how a single medical professional outlines structures on different occasions. MRI's superior soft tissue contrast can help reduce ambiguity for a single observer when outlining structures on multiple scans of the same patient.^3^, ^4^ Treatment planning for radiotherapy has traditionally been based on MRI and CT images. However, recent studies have explored MRI‐only treatment planning, which simplifies the process by eliminating the requirement for more scans.^5^, ^6^, ^7^ This reduces patient's radiation exposure and offers improved soft‐tissue contrast, potentially improving treatment precision in regions like the head and neck, thorax, and abdomen where soft tissues are crucial for target delineation and organ‐at‐risk (OAR) sparing, eliminating the need for separate CT scans.^8^, ^9^, ^10^ The research focuses on generating synthetic CT (sCT) data from MRI, which is cost‐effective, and addresses patient discomfort during imaging procedures. This approach offers potential benefits, including reducing radiation exposure for patients and improving soft‐tissue contrast compared to traditional CT scans. This superior contrast is crucial for accurately defining treatment targets and sparing healthy OARs, particularly in anatomies where soft tissues play a vital role. However, it is acknowledged that sCT may not always lead to an overall improvement in treatment planning due to treatment site dependence, limitations of current techniques, and the need for further validation. Despite these challenges, it is believed that ongoing research will continue to refine sCT methods and solidify its role in radiation therapy planning (RTP).

**FIGURE 1:**
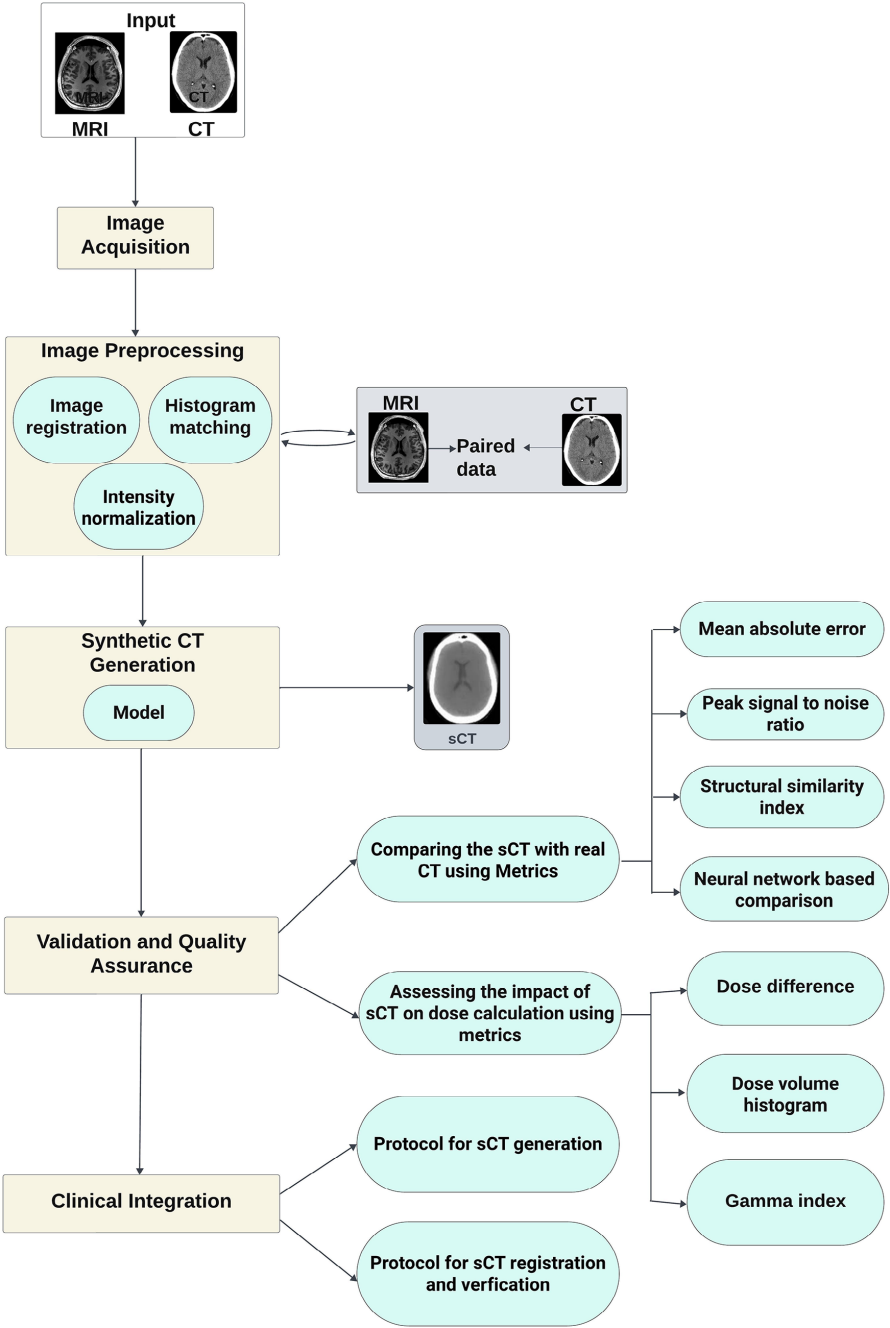
A general workflow for generating sCT from MRI. MRI; Magnetic resonance imaging; sCT, synthetic computed tomography.

In the field of radiation oncology, precise treatment planning and dose calculation are crucial for the successful treatment of cancer patients. Radiotherapy planning currently heavily relies on CT scans for spatial resolution. However, MRI's superior soft‐tissue contrast offers advantages for target delineation. Advancements in sCT generation from MRI data leverage these advantages while minimizing radiation exposure. This could potentially lead to a shift towards MRI‐based workflows in the future.^11^, ^12^, ^13^, ^14^, ^15^, ^16^, ^17^, ^18^, ^19^, ^20^ However, it is difficult to calculate dose when there is no information about electron density in MRIs. A potential fix for this issue is sCT synthesis employing MRI data and several approaches have been investigated in this line of work utilizing a number of techniques. Figure 1 shows a general workflow for generating sCT from MRI scans.

This study comprehensively reviews recent advancements in applying various approaches and techniques to generate sCT from MRI for radiation therapy treatment planning. We delve into the various deep learning (DL) architectures and imaging protocols employed, providing detailed statistics that illuminate trends within the field. Additionally, the paper explores promising applications of sCT for radiation therapy, highlighting its potential impact on different aspects of the treatment process. Finally, we critically evaluate the current state of clinical readiness for these DL‐based sCT generation methods, fostering a discussion on their future implementation in clinical practice.

## 2 METHODS AND MATERIALS

**FIGURE 2:**
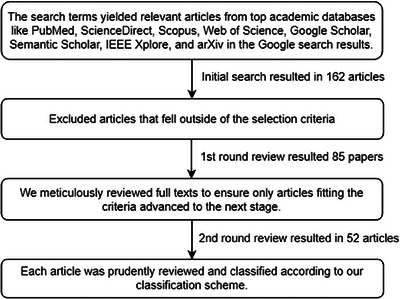
The process of extracting and filtering articles.

The PRISMA^21^ guidelines were used to conduct a systematic review of techniques. We used predetermined criteria to search the relevant literature for each year spanning from 2013 to 2023, using keywords like “synthetic CT from MRI”, “MRI‐only radiotherapy”, “MRI based synthetic CT generation”, and “sCT from MRI in radiation therapy”. Most of the studies are found from the databases of PubMed, Scopus, Google Scholar, Semantic Scholar, IEEE Xplore, ScienceDirect, and arXiv. Articles about producing sCT from MRI for radiotherapy applications were the main focus of the search. Only journal articles and a few highly cited conference papers were selected from the collected literature to be included in the analysis. Book chapters, conference papers with insufficient results, and studies found irrelevant were excluded. Studies meeting these criteria were then categorized under relevant approaches used to generate sCT from MRI. Figure 2 illustrates the steps we followed to filter and extract the relevant articles.

The subsequent sections present the detailed process that we used to extract articles, in addition to our article selection standards and screening procedures.

### Selection criteria

Articles of interest on the creation of sCTs from MRIs were chosen and accepted based on four criteria for additional review. Articles that failed to satisfy the subsequent selection criteria were not accepted:

1. Our review compiled articles exploring the MRI‐based sCT image generation, delving into the diverse tools, software, and methodological approaches employed.
2. The study focuses on recent articles published between 2013 and 2023, highlighting the surge in MRI to CT data conversion methods over the last decade. The transition was initiated and maintained, particularly in the investigation of DL methodologies, which gained momentum in 2016 with the presentation of the first study using DL.^22^
3. The study excluded book chapters, dissertations for master's and doctoral degrees, abstracts, and non‐English articles and only included peer‐reviewed journal articles, as they are commonly used by practitioners and academics to obtain and disseminate research findings.

### Reviewing process

We used three manual screening rounds to find the articles that discussed the methods, tools, or software for generating sCT from MRI. The articles that met our criteria were grouped according to our classification scheme. Here is an overview of our review process:

1. First round: We scanned each article's titles, authors, abstracts, keywords, and conclusions and applied our selection standards. Articles without tools, software, and methods for sCT from MRI as their main topic were not included in our selection. This round reduced the number of articles from 162 to 85.
2. Second round: We read the full texts of the articles and analyzed them based on their central theme. We excluded articles that did not provide sufficient information or relevance to our research question. This round reduced the number of articles from 85 to 52.
3. The final set of articles was then thoroughly read and analyzed according to our classification scheme, which is explained in the next section.

### Classification

**FIGURE 3:**
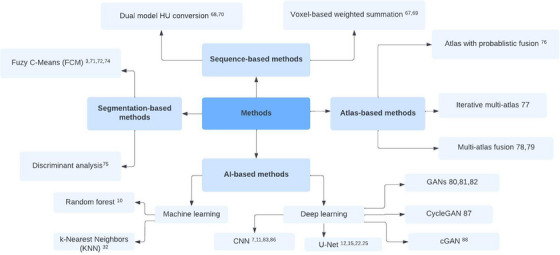
Categories and sub‐categories of approaches for MRI to CT. CT, computed tomography; MRI; Magnetic resonance imaging.

We developed a literature classification scheme to systematically explore and analyze research findings on MRI to CT. This scheme was developed based on the research topics extracted from the 52 articles selected through our filtering process. Figure 3 presents a graphical depiction of the categories and subcategories of the adopted approaches, for generating sCT from MRI, and how they relate to each other. Every study has been categorized into an applied methodology category. We created Tables 2, 3, 4, 5 to 6, to provide a summary of the important information about the approaches for every category. We found most of the approaches falling under Sequence‐, Segmentation‐, Atlas‐, and Artificial Intelligence (AI)‐based methods.

**TABLE 1:** Some market‐ready or undergoing active research MRI‐only systems for sCT.

Company	Solution	Tumor site	Approach	Ref.
Philips	Ingenia MR‐RT XD	Brain, Pelvis, and Prostate	Attenuation	11
Philips	MR for Calculating ATtenuation (MRCAT) H&N	H&N	MRCAT	12
Philips	MRCAT Brain	Brain	MRCAT	13
Philips	MRCAT Pelvis	Pelvis	MRCAT	14
Philips	MRCAT Prostate + Auto‐Contouring	Prostate	MRCAT	15
Siemens Healthineers	syngo.via RT Image Suite	Brain, and Pelvis,	AI	16
Elekta AB / Elekta Unity	MR‐Linac	Brain, H&N, and Pelvis	AI	17
GE Healthcare ‐ Spectronic Medical	MRI Planner	Brain, H&N, and Pelvis	AI	18
ViewRay Inc.	MRIdian	−	Hybrid: atlas‐based + voxel‐based methods	19
MVision AI	MVision	Anatomical regions	AI (DL ‐ GAN)	20

Abbreviations: AI, artificial intelligence; DL, deep learning; GAN, generative adversarial network; H&N, head‐and‐neck; MRI, magnetic resonance imaging; sCT, synthetic computed tomography.

**TABLE 2:** Sequence‐based approaches.

Ref.	Approach	MRI‐seq.	Tumor site	Dataset: No. of patients	Software
23	Voxel‐based weighted summation	T1W	Prostate	9	MATLAB
24	Voxel‐based, weighted summation	T2W, FLAIR and UTE	Brain	10	MATLAB
25	Dual model HU conversion	T1W and T2W	Prostate	10	MATLAB
9	Dual model HU conversion	T1W and T2W	Prostate + Brain	20	MATLAB and CERR

Abbreviation: HU, Hounsfield Unit.

**TABLE 3:** Segmentation‐based approaches.

Ref.	Approach	MRI‐seq.	Tumor site	Dataset: No. of patients	Software
26	FCM	STE and Dixon	Brain	5	MATLAB
27	FCM	dUTE and Dixon	Brain	9	MATLAB
28	FCM	T1W and T2W, Dixon, UTE	Brain	10	MATLAB
28	FCM	T1W and T2W, Dixon, UTE	Brain	10	MATLAB
29	FCM	−	Abdomen	5	Velocity AI, Slicer 4.6
30	Discriminant analysis	dUTE and T2w	Brain	3	MATLAB, TRiP98

Abbreviations: AI, artificial intelligence; FCM, fuzzy c‐means clustering.

**TABLE 4:** Atlas‐based approaches.

Ref.	Approach	MRI‐seq.	Tumor site	Dataset: No. of patients	Software
31	Atlas‐based regression	T1	Head region	10	−
32	Iterative multi‐atlas CT synthesis	T1	Neck	13	NiftyReg, NiftSeg, NiftySim
10	Multi‐Atlas	T2	H&N, Prostate	21	Plastimatch
33	Multi‐Atlas	T2	Prostate	39	Eclipse, Varian Eclipse

**TABLE 5:** AI‐based approaches.

Ref.	Approach	MRI‐seq.	Tumor site	No. of patients	Software
34	Sup. ML: KNN regression	−	Brain	13	−
35	Sup. ML: Random forest	−	Head	14	−
36	Sup. ML: Deep Boosted Regression	T1 and T2	Brain	20	NiftyNet, TensorFlow
37	DL: GANs	T1	Brain	15	SPM 12
38	DL: GANs	T1 & T1c & FLAIR	Brain	ADNI: 3,416 pairs of T1W MRI, BRATS: 274	Python (NiftyNet, TensorFlow, Keras)
39	DL: GANs	T1 & T2 & T2‐FALIR & T1‐Gd	Brain	220	Python (TensorFlow)
40	DL: CNN	UTE	Brain	7	Python (TensorFlow)
41	DL: CycleGAN	T1 & T2	Brain	24	Python (TensorFlow)
42	DL: FCN	T1	Brain and pelvic	−	Python (TensorFlow)
43	DL: CNN	T1	Prostate	20	Python (TensorFlow)
44	DL: cGAN	Dixon	Prostate, rectal, cervical	91	Python (TensorFlow)
45	DL: U‐Net NN	−	Intracranial	60	dipy library to align MR and CT
46	DL: DualGAN	T1	Brain	78	−
47	DL	−	Brain and pelvis	13	−
5	DL: a residual learning based u‐shaped deep neural network (RUN)	T1/T2	Brain	35	Software: Keras library.
48	DL: CNN	−	Brain	60	−
49	DL: GAN and ResNet	−	Brain	86	−
50	DL	T1	Sacroiliac joints	30	−
51	DL: CNN	−	H&N	44	TFE algorithm
52	DL: U‐Net, residual U‐Net, GAN, residual GAN	−	H&N	23	−
53	DL: GAN	−	intracranial tumor	31	TensorFlow 2.4.1
54	DL	−	H&N	39	−
6	DL: 3D U‐Net DL	−	H&N	120	MIM software, PyTorch
55	DL: 2D CNN‐UNet	−	Brain	54	Tensorflow, Nadam
56	DL	T1 or T2	Pelvis	26	−
57	DL: RTCGAN	−	Pelvic	19	pytorch, Adam
58	DL: a patch‐based neural network	−	Hip, knee and ankle	131	−
55	DL: multi‐task 2D CNN U‐Net	−	Brain, ROI: bone	54	−
59	DL: Unet, CycleGAN		Whole body	127	MIM software
60	DL	−	Nasophary‐ ngeal carcinoma; H&N	78	−
61	DL	T1, T2	Nasophary‐ ngeal carcinoma	32	−
62	Hhybrid deep network: hybrid multi‐scale synthesis network (HMSS‐Net)	−	H&N	78	pix2pixHD model software
63	Hybrid deep network	T1, T2	H&N	90	pix2pixHD model software

Abbreviations: CNN, convolutional neural network; DL, deep learning; GAN, generative adversarial network; ML, machine learning.

**TABLE 6:** Hybrid approaches.

Ref.	Approach	MRI‐seq.	Tumor site	No. of patients	Software
64	Bulk density assignment, tissue class density assignment, hybrid multi‐atlas, and DL	−	Pelvic	40	MATLAB, Eclipse TPS (Varian Medical Systems)
65	MRI‐to‐CT denoising diffusion model (MC‐DDPM)	−	Brain and prostate	64	PyTorch
66	Pattern recognition and atlas registration based	T1W	Brain	17	MATLAB
33	Multi‐atlas local weighted voting, a structure‐guided deformable registration, and symmetric rigid registration based	T1W and T2W	Prostate	39	MATLAB
67	Commercial solutions with different methodologies	−	Brain and pelvis	94	−

**FIGURE 4:**
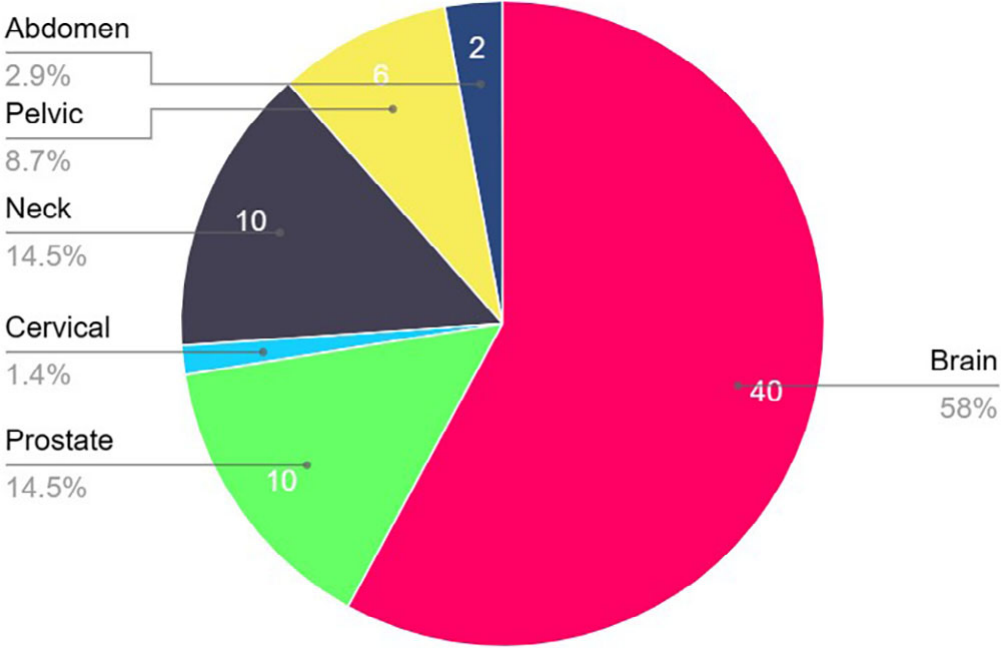
Number of studies focused on a body region/specific cancer tumor.

Figure 4 presents the distribution of studies across different tumor sites, with brain, H&N, prostate, pelvis, and abdomen being among the most investigated.

In addition to sequence, segmentation, atlas, and hybrid‐based approaches for image synthesis,^35^ a number of the latest methodologies have utilized machine learning (ML) techniques like random forest and dictionary learning based methods, also DL models including convolutional neural networks (CNNs) and generative adversarial networks (GANs). In clinical and research contexts, GAN and residual networks (ResNets) are widely used for MRI‐only RTP and MRI‐guided positron emission tomography (PET) attenuation correction. These models are especially interesting because of their promising performance and ability to generate sCT images from MRI data.^49^ Subsequent subsections present the overview of some conducted research work under prominent categories of the techniques used to generate sCT.

### 2.1 Sequence‐based techniques

Sequence‐based methods for sCT generation use variations in signal intensity captured by different MRI sequences like T1 weighted images (T1W), T2 weighted images (T2W), and dixon etc.^1^, ^68^ These variations often correspond to differences in tissue properties. By analyzing these variations across multiple sequences, they can estimate Hounsfield Unit (HU) values for each tissue type within the patient's anatomy. This approach offers several advantages, including not requiring pre‐existing CT scans for training and directly estimating electron density based on MRI information, which can create sCT images that match the patient's specific anatomy for accurate dose calculation in radiotherapy planning.

Several studies have used sequence‐based methods to generate sCT images for various cancer and radiation modalities. The method of voxel‐based weighted summation was employed to convert HU from MRI intensities and create sCT images. The assessment of the sCT images against conventional CT scans was done by measuring HU discrepancies, dose reduction ratio (DRR) concordance, and dose profiles. The sCT images were suitable for accurate treatment design and patient alignment with negligible errors.^23^, ^24^

To produce pseudo‐CT images from MRI, a dual model HU conversion method employed different models depending on whether the MRI intensity values are either outside or inside the bone segment. The technique made use of a linear regression model for the soft tissue and a polynomial regression model for the bone. The technique was verified by using phantom and patient data and comparing it with a single model HU conversion technique. The technique showed improved accuracy of sCT images and reduced dosimetric errors in the treatment planning process.^9^, ^25^

### 2.2 Segmentation‐based methods

Segmentation‐based methods for generating sCT use image segmentation techniques. Through this process, the MRI image is effectively divided into distinct regions that represent different tissue types.^69^, ^70^ Next, a CT value is assigned to each kind of tissue, either using statistical models or pre‐existing knowledge about tissue properties. This method provides a more straightforward means of converting MRI data into CT‐like data.

A segmentation‐based method, called fuzzy c‐means clustering (FCM), segmented MRI images into different tissue types based on their MRI intensities or phase values. Then, it assigned CT values to each tissue type using some prior information, such as probability, linear combination, or predefined values.^26^, ^27^, ^28^ Using different MRI contrasts, FCM created sCT images that can be used within a spatial constraint. The technique^28^ generated the sCT from several MRI contrasts by adding each voxel's attenuation characteristics.

Another approach^27^ used a single‐acquisition, under‐sampled UTE‐mDixon pulse sequence that can capture both bone information and soft tissue in a short time. The method performed unsupervised clustering on the images to segment the brain into five tissue types and produced pseudo‐CT images by linear combination.

A four‐class μ‐map was generated to address PET data attenuation in the head region utilizing MRI.^26^ The method's effectiveness was assessed by comparing the MRI‐derived μ−‐map with the corresponding CT‐derived map, and both techniques were employed to correct the PET images.

In another study, a different technique was used to retrieve pseudo CT values from MRI scans. The method utilized a statistical classification technique called discriminant analysis and applied it to create and adjust ion radiation therapy plans for head‐and‐neck (H&N) tumor patients. The method aimed to reduce errors due to anatomical variations and improve the accuracy of dose delivery.^30^

### 2.3 Atlas‐based methods

Atlas‐based methods for sCT generation make use of an already‐existing database of paired MRI‐CT scans.^64^ This database serves as a reference, or “atlas”, linking the anatomical structures evident in MRI images to their corresponding HU values in CT scans. To discover the best match between patient's MRI scan and comparable scans in the atlas, the technique essentially uses deformable image registration techniques. The corresponding voxel in the most closely matched atlas CT scan is used to assign a CT number to each voxel in the patient's MRI based on this alignment. These techniques have increased the accuracy of sCT; nonetheless, they are constrained by the requirement for a vast and varied database, as well as possible errors in the registration procedure itself.^71^

Different atlas‐based techniques to produce sCT images from MRI images for various applications in radiotherapy were presented.^10^, ^31^, ^32^, ^33^ Multiple atlas fusion, patch‐based matching, single‐atlas warping, and probabilistic modeling are among the categories of atlas‐based techniques for creating sCT from MRI that provide varying approaches to tailor anatomical templates to specific scans. These methods differ in some aspects, such as the type of MRI images, the region of interest (RoI), the number of atlases, and the additional steps to improve the sCT quality.

A study used atlas‐based regression to produce customized head pseudo‐CTs for patients from their MRI data.^31^ The method used a database of paired scans and deformable registration techniques to warp CTs onto the target image. The model predicted pseudo‐CT values for each voxel, suggesting atlas‐based regression held promise for RTP. A multi‐atlas based approach used multiple atlases (CT and MRI scans of the H&N or the prostate) that are selected and combined to generate the sCT image of the corresponding region.^10^ The approach did not use any additional steps to improve the sCT quality. Another technique was proposed for generating H&N sCT images from paired MRI and CT data^32^ and this technique iteratively selected and combined the most relevant atlases for each target image, followed by a bone refinement step to enhance sCT quality. A multi‐atlas local weighted voting approach was proposed to analyze pelvic MRI and CT images.^33^ The local resemblance of the atlases to the target MRI picture determines which ones were used. Three small field‐of‐view images were used for generation of sCT images and outline prostate contours.

### 2.4 AI‐based methods

AI is revolutionizing the field of sCT generation from MRI data.^72^ AI algorithms are trained on vast datasets of paired MRI‐CT scans, learning the complex relationships between MRI characteristics (such as signal intensity and texture) and the corresponding tissue densities represented by HU in CT scans. Two main AI approaches for sCT generation are ML and DL. ML uses algorithms like Random Forests to map features in MRI images to corresponding CT HU values, while DL uses GANs, which involve two competing networks: a generator that creates realistic sCT images from MRI data and a discriminator that differentiates between real CT scans and generated ones. These methods offer a powerful and ever‐evolving approach to sCT generation, potentially surpassing traditional techniques in terms of accuracy and generalizability, leading to advancements in medical imaging and treatment planning.

Enhancements to medical image analysis, such as denoising, image segmentation, generating pseudo‐CTs from MR scans, and reconstruction are being made possible by ML, specifically CNN.^37^, ^73^ Given their reliability, precision, and robust performance, a limited set of DL models is typically utilized. Among the DL models used for sCT generation, GAN and ResNets have demonstrated exceptional performance, particularly in image transformation and segmentation tasks related to medical image analysis.^74^, ^75^, ^76^, ^77^, ^78^, ^79^

#### 2.4.1 ML‐based methods

A ML‐based technique was presented to sCT image generation from traditional T1W MRIs using anatomical signatures inside a random forest framework.^35^ This technique used an iterative refinement model with a classification random forest to produce semantic information. To enhance one‐to‐one MRI‐CT mapping, the semantic information was incorporated into the regression random forests‐based technique. Another approach was proposed employing feature matching and nonlinear local descriptors to predict MRI‐based pseudo CT images.^34^ Feature map, supervised manifold regularization, and low‐rank approximation were used in this method. A DL‐based method for synthesizing sCT images from MRI was presented for abdominal radiotherapy treatment planning.^29^ The authors classified MRI images into five tissue types: fat, air, spine, lungs, and high‐density tissue, employing FCM and population‐based HUs. The sCT images were evaluated on a dataset of five volunteers.

#### 2.4.2 DL‐based methods

In a review,^1^ various DL methods including the first published study in 2016 that used a DL method to generate sCTs from MRIs in MRI‐only radiotherapy were discussed, overviewing different networks, loss functions, and image and dose evaluation results. Two main DL architectures are GANs and generator‐only networks. Generator‐only networks are fairly simple architecture, consisting of a single component (neural network), commonly a CNN, that directly maps the features of an MRI image to those of a CT image. GANs are more complex but can produce more realistic sCT images. They are made up of two neural networks, a discriminator network and a generating network. The generator network produces a sCT image from an MRI scan, while the discriminator network finds out whether the image is a synthetic or a real CT image. The training procedure involves an adversarial process between the two networks: the discriminator network aims to enhance its ability to discriminate between genuine and sCT images, while the generator network attempts to trick the discriminator network into thinking that its sCT images are real. Up to now, numerous studies have been made based on DL methods that offer a spectrum of approaches for SCT generation for several anatomical localizations.^35^, ^49^, ^64^ The authors concluded that DL methods generate high‐accuracy sCTs, are flexible, and can be adapted to different anatomical regions and imaging protocols. They also highlighted their speed, making them suitable for clinical use. From GANs to U‐Net, 29 DLM studies were selected to include in the study meeting the requirements and are discussed under their relevant DL architecture for sCT generation, given as below:

##### Generative adversarial network (GANs)

GANs have been used for a variety of purposes and goals to produce artificial data that is identical to real data.^1^ It has been shown in different studies that sCT images created with GANs are more realistic than those created with other techniques. A DL‐based method utilized DualGAN architecture for synthesizing sCT images from MRI for radiotherapy treatment planning.^46^ The sCT images showed a mean‐absolute‐error (MAE) of 61 HU and preserved soft tissue contrast features for precision tumor alignment. The method aimed to eliminate the need of CT scans especially for patients who can not undergo CT scans. However, it required a large dataset for training and validation. A review presented an overview of DL methods for generating sCT images, which have clinical applications in radiotherapy and other medical imaging fields.^68^ The performance of sCT generation on various datasets was evaluated and it is found that DL methods including GANs, variational autoencoders (VAEs), and cycle‐consistent GANs (CycleGANs) can generate high‐quality sCT images. Beyond just simply producing sCTs that are visually realistic, GANs hold great potential. It is reported that GAN‐based frameworks are being investigated by researchers for applications such as tissue segmentation for diagnosis, radiation dose calculation, and even personalised sCT generation for individual patients. GANs face challenges in sCT generation due to the need for large, high‐quality MRI datasets, achieving pixel‐perfect accuracy in HU, and overcoming anatomical details and tissue misclassifications. Careful data acquisition, rigorous validation, and interpretability methods are crucial for their full potential. Their future directions included developing more efficient models and integrating sCT generation with other imaging modalities such as enhancing multimodal imaging capabilities and integrating advanced technologies like PET for comprehensive and synergistic medical imaging solutions. A work on a novel Residual Transformer Conditional Generative Adversarial Network (RTCGAN) was carried out for MRI‐based CT synthesis in the pelvic region.^57^ The network used a hybrid CNN‐Transformer structure to save global picture context and local texture details. The outcomes of the experiments revealed that RTCGAN surpasses the most recent methods in image quality metrics, preserving anatomical structures and reducing artifacts, indicating its potential in the context of clinical practice.

##### U‐Net architecture and variants

A popular convolutional encoder‐decoder architecture for sCT generation is called U‐Net. An encoder in the U‐Net gathers features from the MRI image, while a decoder reconstructs the sCT image. High‐quality sCT images are demonstrated to be produced by the U‐Net. A DL‐based method was presented for synthesizing sCT images from MRI for intracranial tumor radiotherapy treatment planning.^45^ The method utilized a U‐Net architecture and had a MAE of 17.6 HU in soft tissue. The authors discussed challenges and potential benefits, including reducing CT scans and addressing differences in image intensity distributions between MRI and CT. Another study explored the use of DL to create sCT images from PET and MRI data for RTP in H&N cancer patients.^52^ Four DL models were compared: U‐Net, residual U‐Net, GAN, and residual GAN. The residual GAN model was found to be the most accurate, with the lowest MAE and highest peak signal‐to‐noise ratio (PSNR). The study concluded that residual GAN is a promising approach for sCT generation. A U‐Net‐based DL model for H&N sCT from bone MRI was developed that can be utilized to construct sCT scans from bone MRI images.^54^ A dataset of 39 adults and paediatric patients with H&N CT and bone MR imaging was used to train the model. Three encoder‐decoder models were trained: Light_U‐Net (2 million parameters) and VGG‐16 U‐Net (29 million parameters) both with and without transfer learning. VGG‐16 models were surpassed by the Light_U‐Net architecture in terms of bone precision and recall, with performance of Light_U‐Net increasing after adding local training data to external sources. According to the authors' conclusion, their methodology showed great potential in easing the integration of clinical application and downstream image processing. A study proposed a method for accurate bone density value prediction by splitting global sCT value regression into RoI HU regression tasks and RoI anatomical segmentation.^55^ Following U‐Net‐like design, it is discussed that the approach can be optimized using a composite loss function and is network‐adaptable. The method compared RTP dosage calculation maps from CT and sCT, and its simplicity and dependability make it appropriate for broader clinical studies. Another Unet and CycleGAN models based approach was proposed to generate MRI‐to‐CT images, evaluating image quality and radiomic features.^59^ Findings only displayed a small percentage of the attributes showing good/excellent similarity. In terms of image quality measures (IQMs), Unet‐sCT outperformed CycleGAN‐sCT. But in terms of radiomic characteristics, neither showed complete superiority, and both were significantly less comparable to actual CT, suggesting current DL methods cannot effectively learn target image radiomic features. Therefore, it was stated that enhancing the radiomic resemblance for MRI‐to‐CT generation will take more effort.

##### Residual Neural Networks (ResNets)

ResNets are a type of DL architecture that mitigate the vanishing gradient problem by using residual connections to make training very deep neural networks easier. By introducing shortcuts that skip over some layers and allow information to flow directly, ResNets overcome the drawbacks of deep neural networks, improving accuracy and performance. For the purpose of MRI based CT generation, a residual learning‐based u‐shaped deep neural network (RUN) was presented.^5^ The network, consisting of 34 convolutional layers, learned the mapping between CT and MRI scans, generating sCT images comparable to original images regarding accuracy and quality. The Adam optimizer was employed to train the network and evaluated on a test set of paired CT and MR images. The results showed high accuracy and quality, making the RUN network a promising method for radiotherapy planning and other applications. A comparison was made and the performance of two DL models, ResNets and GAN was presented, for sCT generation from MR images.^49^ Analysis was done on 86 participants' brain MR and CT scans. The ResNet model showed higher accuracy in delineating brain tissues, estimated CT scan results for the complete head, and offered comparable Structural Similarity Index Measure (SSIM) and PSNR metrics. The ResNet model demonstrated potential for generating sCT in MRI‐only RTP and PET/MR attenuation correction.

##### Convolutional Neural Network (CNN)

A CNN framework was employed to develop a DL‐based method that generates sCT images from MRI for pediatric brain radiotherapy planning, with the goal of minimizing the MAE between the real CT and synthetic images.^48^ The CNN generated high‐accuracy sCT images, which are then used to calculate dose distributions for photon and proton radiotherapy. The study demonstrated the method's potential for pediatric brain radiotherapy planning. A CNN‐based method for generating sCT images from MRI data, utilized a three‐dimensional spoiled dual‐echo gradient‐echo pulse sequence that is T1W to acquire the MRI data.^50^ The sCT images were generated using a CNN that is trained on a dataset of paired CT and MRI images. The method was evaluated on a dataset of 30 patients with suspected sacroiliitis. The sCT images are compared to the original CT images using a variety of metrics, including the detection rate of erosions, sclerosis, and ankylosis. It was found that the sCT images have a higher detection rate for erosions and sclerosis than the MRI images, and a similar detection rate for ankylosis. The authors concluded that the DL method they developed can be used to generate sCT images with promising accuracy for the detection of sacroiliitis. A new method for generating sCT from zero‐echo‐time (ZTE) MRI images, used a multi‐task CNN to estimate CT‐like HU values, segment the bone region, and classified bone density.^55^ The method was evaluated on a dataset of 54 brain patients with paired ZTE‐MR and CT images. Another kind of CNN that was employed for sCT generation was the deep CNN (DCNN). Several convolutional layers make up the DCNN, which uses the MRI image to extract features. It was demonstrated that sCT images generated by the DCNN resemble actual CT images. A 3D DCNN was proposed to generate synthetic megavoltage CT (sMVCT) for MRI‐only radiotherapy treatment planning.^6^ The network was trained on 120 H&N cancer patients, and the results showed similarity to real MVCT images and dose distributions, suggesting sMVCT can be effective for MRI‐only radiotherapy treatment planning. A commercial CNN‐based method was used to convert MRI to H&N sCT, resulting in statistically equivalent and accurate absorbed dose calculations in comparison with CT‐based radiotherapy. A study involving 44 patients with H&N cancer found that sCT data can replace CT data in MRI‐only radiotherapy workflows, with MAE of 0.30% for all dose‐volume histograms. A commercial CNN based method was used to convert MRI to H&N sCT and it is reported that the absorbed dose estimations were performed with accuracy and statistical equivalency when compared to CT‐based radiotherapy.^51^

##### Other DL Architectures

This section explores approaches that are deemed hybrid, as they do not readily fit into any of the previously mentioned specific categories of DL methods rather they employ some DL architecture with other traditional methods. It was revealed in a study that the choice of input MRI sequences significantly impacts the quality of sCT generated using DL.^56^ Four different MRI protocols were compared: Dixon MRI, conventional T1W or T2W MRI, ZTE MRI, and UTE MRI (RESOLUTE). Dixon MRI was found to be adequate for generating quantitatively precise sCT images, while ZTE MRI improved the capture of bowel air distributions. The findings suggested that input MRI sequences should be tailored to specific applications and desired characteristics. Another work made use of GAN and pixel‐to‐pixel(pix2pix) model for synthesizing sCT images from MRI for intracranial tumor radiotherapy treatment planning.^53^ Model was trained using a patch‐based dataset and performance of the model got improved through data augmentation. A dataset comprising 31 participants was used to assess the model, with 26 pairs used for training and 5 pairs for testing. It was reported that the sCT images showed high similarity to real CT images. Two radiologists independently assessed the quality of sCT images, evaluating spatial geometry, noise level, contrast, artifacts, and imaging details and expressed their excellent satisfaction. A DL model was explored for generating high‐resolution sCT images from MRI for orthopedic applications.^58^ The model, trained on MRI and CT images from hip, knee, and ankle regions, was found to generate accurate sCT images with comparable MAE and PSNR assessment metrics. A study evaluating four methods for generating sCT in MRI‐only definitive radiation to the pelvis, in both male and female patients, found that all four methods demonstrated comparable dosimetric accuracy for organising target volume and organ structures that are at danger.^64^ Whereas the tissue class density assignment method divided tissue into three classes, the bulk density assignment approach awarded a single HU value to the entire volume. The hybrid multi‐atlas method compared the CT and MRI pairings that are co‐registered in a library, taking tissue in homogeneity into account. The DL method used a CNN to convert MRI into sCT and it had shown the dose difference with the lowest median. The authors concluded that all four sCT generation techniques could be applied with similar dosimetric accuracy for MRI‐only definitive pelvic radiation, with factors such as cost, image guidance considerations, and implementation ease influencing the choice of approach. Within the framework of abdominal radiotherapy treatment planning, a study^29^ introduced a DL‐based approach for generating sCT images from MRI data. The methodology involved the classification of MRI images into five distinct tissue types‐air, lungs, fat, spine, and high‐density tissue‐utilizing FCM and population‐based HUs. The sCT images were evaluated on a dataset of five volunteers and were found to have a MAE of 3–29.1 mm for air, 0.4–1 mm for lungs, 0.5–1.3 mm for fat, 1.2–1.4 mm for spine, and 0.7–1.6 mm for high‐density tissue. A white paper discussed the sCT use for MRI‐only radiotherapy planning for the brain and pelvis.^47^ The workflow involved a patient's MR scan, sCT generation, OARs‐any organs or tissues near the tumor target area and contouring of target volume, and exporting to a method for calculating doses in treatment planning. Clinical validation showed sCT can generate dose distributions as accurately as CT scans. The reported key benefits included improved soft tissue contrast, reduced radiation exposure, and increased patient convenience. A 3D transformer‐based denoising diffusion probabilistic model (MC‐DDPM) is proposed for MRI‐based sCT image generation.^65^ The model adds Gaussian noise to real CT images and denoises noisy CT images from MRI scans. The efficacy of this approach is demonstrated by experiments conducted on T1W MRI‐CT datasets of the prostate and brain. However, because of its lengthy Markov chain of diffusion phases, MC‐DDPM is computationally expensive. Another study compared four methods for generating sCT images from MRI images, focusing on geometric accuracy and preservation of original CT values.^67^ The four methods included Syngo BD, Spectronic, Syngo_AI, and Therapanacea, with all showing promising results, but the choice depends on the specific application.

## 3 APPLICATIONS OF SCT

The use of sCT in medical imaging is broad and includes MRI‐only workflows for radiotherapy treatment planning, PET attenuation correction, image‐guided adaptive radiotherapy (IGRT), and more.^35^ It is a useful substitute for traditional CT scans, providing advantages like lower cost, reduced radiation exposure, and more patient convenience by presenting a simplified workflow, improved soft tissue visualization, and personalized planning. Following is brief elaboration of some promising applications of sCT for radiation therapy:

### Personalizing treatment plans

sCT is a promising tool for personalized radiotherapy treatment plans, enhancing outcomes and reducing side effects in cancer patients.^7^, ^80^ sCT data can be used to create computational models that simulate individual patient responses to radiation therapy, predicting tumor shrinkage, potential complications, and optimal dose distribution. In other words, sCT is used in patient‐specific models for precise tumor localization, facilitating precise radiation therapy delivery to the tumor site, and adaptive radiotherapy. It provides real‐time information on tissue changes, ensuring optimal tumor targeting and minimizing radiation exposure to healthy tissues.

### MRI‐only radiotherapy

MRI‐only radiotherapy is a treatment method that uses MRI for tumor visualization and treatment planning, eliminating the need for traditional CT scans.^81^ This approach uses MRI's superior soft tissue contrast to accurately delineate tumors and OARs, potentially improving treatment outcomes. MRI‐only radiotherapy has several benefits, including improved target delineation, reduced radiation exposure, enhanced patient comfort, and a simplified workflow. Current applications include treatment for prostate cancer because of its excellent soft tissue contrast, H&N cancer for precise tumor delineation, and lung, rectal, and brain tumors. However, challenges include the development of sCT technology, limited availability of MRI centers, and regulatory hurdles.

### PET attenuation correction

PET attenuation correction is crucial for accurate PET image interpretation and quantification. Traditional CT‐based attenuation correction uses x‐ray CT scans, but they involve additional radiation exposure and are not always available in all treatment settings. sCT offers a promising alternative by generating a pseudo‐CT scan from patient's PET or MRI data.^82^ This image can be used for attenuation correction on PET images, reducing radiation exposure, improving patient comfort, and enhancing workflow. sCT can be utilized for MR‐based treatment planning, adaptive radiotherapy, and PET‐based treatment response assessment. However, sCT is still under development and its accuracy and efficacy are not fully established. Further research is needed to optimize sCT algorithms and ensure clinical validation.

### Conversion of cone‐beam computed tomography (CBCT) to CT

CBCT is a specialized imaging method applied in dental and H&N imaging. However, CBCT images have lower resolution and higher noise compared to CT scans. This is where sCT comes in. sCT which uses advanced algorithms, converts CBCT images into CT‐like images, improving image quality and reducing radiation exposure.^83^, ^84^ This process streamlines workflow, eliminates the need for separate CT acquisitions, and enhances diagnosis and RTP in various applications. sCT can visualize tumors and surrounding tissues, improve dental procedures, and assess bone structures for orthopedic surgeries. It also shortens patient imaging times, improving their overall experience. CBCT to sCT offers a viable strategy to raise the image quality, reduction in radiation exposure, and streamlining clinical workflow, potentially revolutionizing medical applications and improving patient care and outcomes.

### Clinical Commercial Software available for generating sCT

Several companies and research institutions are actively working on creating MRI‐only systems or investigating ways to produce sCT from MRI data. Among the well‐known companies are Varian Medical Systems (bought by Siemens Healthineers), ViewRay Inc., GE Healthcare, Siemens Healthineers, and Philips Healthcare. Some of their commercially available or under active research MRI‐only systems for sCT generation are listed in Table 1, obtained from official websites of the respective companies, and industry news articles.

## 4 RESULTS

**FIGURE 5:**
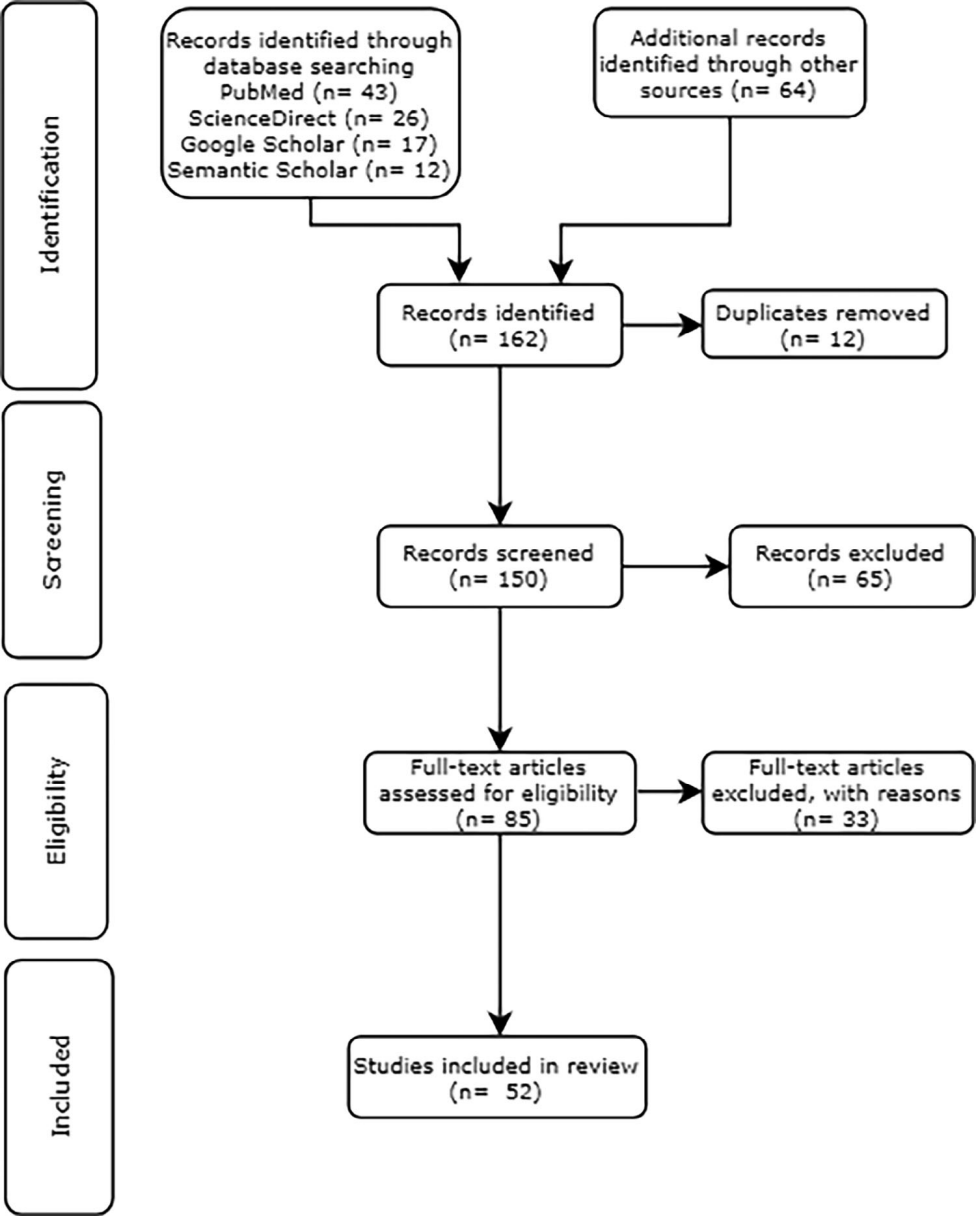
PRISMA flow diagram for the selection of studies included in the review.

A database search on PubMed, Science Direct, Google Scholar, Semantic Scholar, IEEE Xplore, arXiv, Wiley online, and Frontiers produced 162 results. After duplicates were eliminated and the content was reviewed, about fifty papers were found to be eligible to be included in the review. Figure 5 presents the number of studies identified, screened, excluded, and included in the review, using a PRISMA flow diagram while the above‐given Figure 4 shows the overall number of publications categorized by the tumor sites taken into account in the studies that were reviewed.

Tables 2, 3, 4, 5 to 6 presents some highlights of the adopted approaches to generate sCT from MRI, in a concise manner.

## 5 DISCUSSION

MRI‐only treatment planning can improve target delineation accuracy because it provides better soft tissue contrast than CT in radiation therapy.^8^ MRI can resolve tumor boundaries, identify regions with a high tumor burden, and provide precise delineation for prostate, uterus, and cervix. Benefits include enhanced dosimetry and the possibility of raising the therapeutic ratio. Currently, CT‐based treatment planning workflow introduces geometrical uncertainties, which can compromise tumor control. MRI‐simulation platforms offer an alternative by generating accurate anatomical patient models in treatment positions. The idea of MRI‐only treatment planning resulted from this, in which the MRI scan is used to provide synthetic or sCT data that is used to calculate dosages. This approach reduces CT scanning, streamlines clinical effectiveness, and fully exploits the advantages of MRI for highly accurate treatment planning.

The reviewed studies adopted diverse approaches to MR image sequences, with some utilizing single sequences and others incorporating multiple sequences. A single MRI sequence is a set of images acquired using a specific imaging protocol. Different sequences are used to highlight tissue characteristics, providing information about the anatomy and pathology of the imaged area. Common types include T1W imaging, T2W imaging, proton density imaging, FLAIR, diffusion‐weighted imaging, and Gradient Echo imaging. The choice of a single MR image sequence depends on clinical or research objectives, and radiologists often use a combination of sequences to obtain a comprehensive understanding of the imaged anatomy and detect and characterize various pathologies. Multiparametric MRI is an imaging technique that uses multiple sequences to provide a comprehensive view of tissue properties. This approach enhances information, improves diagnostic accuracy, aids in personalized treatment planning, and reduces scan times. Common sequences include T1W, T2W, contrast‐enhanced, and diffusion‐weighted. However, multiparametric scans can be longer, more complex, and more expensive. They are used in oncology, neurology, musculoskeletal imaging, and cardiovascular imaging. While it has limitations, its benefits often outweigh the drawbacks, making it a valuable tool in various clinical applications. The technology is used in cancer diagnosis, stroke, epilepsy, neurodegenerative diseases, musculoskeletal imaging, and cardiovascular imaging. In real‐world applications, it is reported that a single MR image sequence is adequate for sCT prediction when speed is the goal. On the other hand, multi‐sequences of MRI are appropriate for achieving accuracy. The optimal approach to sCT generation depends on the specific clinical application and priorities. For routine applications, a single sequence is preferred for simplicity and cost‐effectiveness, while for critical applications, multiple sequences are preferred for superior quality and robustness. Balancing factors like scan time, cost, image quality, and robustness, as well as additional technical expertise, are crucial.

Overall, significant improvements in sCT generation through the application of ML and DL approaches were noted in this review paper. With respect to RTP, it might become more accurate and efficient as a result of these developments by:

1. Reducing the need for conventional CT scans, which may be costly, time‐consuming, and expose patients to radiation.
2. Permitting the use of MRI‐based treatment planning for anatomical areas, such as H&N, where CT scans are often restricted.
3. Enabling the use of patient‐specific anatomical data from MRI for establishing personalized treatment plans.

More detailed discussion about identified advantages and limitations of each reviewed technique is provided below: The benefits and drawbacks of several methods pertinent to generation of sCT from MRI images are covered in this section. For researchers and medical professionals to properly choose the best technique for a certain application, they must be aware of these advantages and disadvantages. It is probable that the section may delve into topics such as precision, effectiveness, expense, intricacy, and suitability for various situations. A comparison of these techniques' in terms of their advantages and limitations is further detailed in Table 7, which summarizes key metrics like accuracy and processing time. Readers can learn a great deal about the trade‐offs involved with each strategy by comparing these factors.

**TABLE 7:** Advantages and limitations of the employed techniques.

	Advantages	Limitations
Sequence‐based methods	‐ Simple and easy to integrate into clinical procedures ‐ Dispense along with extra registrations	‐ Sensitive to noise, artifacts, and variations in MRI sequences ‐ Time‐consuming, and patient discomfort
Segmentation‐based methods	‐ Ability to improve the HU estimation in regions with high contrast	‐ Require manual or semi‐automatic segmentation ‐Time‐consuming and operator dependent
Atlas‐based approaches	‐ Robustness and high accuracy in HU estimation	‐ Computationally intensive ‐ Require a large and diverse database of atlases
DL methods	‐ Improved dosimetric accuracy, faster imaging and workflow, flexibility and adaptability, potential for personalized medicine	‐ Computational resource requirements, extensive annotated datasets are required, restricted generalization, explainability and interpretability

Abbreviations: DL, deep learning; HU, Hounsfield Unit; MRI, magnetic resonance imaging.

### 5.1 Sequence‐based methods

#### Advantages

The advantages of sequence‐based approaches are that they can be easily integrated into clinical workflows, and they do not need any extra information or registrations. This way, they can lower the reliance on complicated image registration and segmentation algorithms, which can cause mistakes and distortions.^85^

#### Limitations

The limitations of sequence‐based methods are that they require MR images from many sequence acquisitions, which can cause discomfort for the patient, increase scanning time, and motion artifacts.^65^ They are also influenced by the MRI sequences employed, the image quality, and the variation in HU values between tissues and patients. They may have difficulties identifying the difference between bone and soft tissue.^85^, ^86^

### 5.2 Segmentation‐based methods

#### Advantages

They can reduce the artifacts and noise in the sCT images. They can provide consistent HU values for the same tissue class across different patients.

#### Limitations

Because of the resemblance between bone and soft tissue in MRI, the accuracy of these approaches depend on the segmentation algorithm, which is time‐consuming and operator‐dependent.^65^ They may not capture the continuous HU variations within each tissue class

### 5.3 Atlas‐based methods

#### Advantages

Atlas‐based approaches can generate high quality sCT images with good accuracy. They have been successfully applied in the clinic and are also being utilized in commercial hybrid sCT generating devices for bone definition.^31^, ^87^

#### Limitations

Atlas‐based approaches use registration of deformable images, but their efficacy is dependent on the accuracy of the registration algorithm.^65^ The capacity of atlas‐based approaches to deal with patients with anatomical abnormalities limits them. They are difficult to generalize and validate since they rely on the availability of atlases for various anatomical areas and populations of patients.^64^

### 5.4 Deep learning strategies

#### Advantages

There are many benefits to sCT generating using DL techniques. Compared to conventional techniques like atlas‐based registration, DL techniques can yield sCT images that are more realistic and accurate which may increase their suitability for clinical applications.

#### Limitations

There are still a lot of issues that need to be resolved even though DL techniques for sCT generation have been successful. DL methods for generating sCT images face challenges like data quality sensitivity, computational cost, and artifact sensitivity, potentially resulting in inaccurate or unrealistic images. For a clearer understanding, these issues are elaborated based on the following:

1. Data quality: The quality of the training data has a major impact on the accuracy of sCT images. The resulting sCT images could be erroneous or unrealistic if the quality of the training data is low.
2. Computation cost: Training and using DL methods can be computationally demanding. Utilizing them in clinical practice may be challenging as a result.
3. Generalization: The degree to which DL models will generalize to new datasets is not always evident because they are trained on a particular dataset of CT and MRI scans. If the training and test (clinical) dataset differ, this could be an issue.

All things considered, the generation of sCT images for radiotherapy could be completely transformed by DL techniques. To overcome the difficulties in using DL techniques, such as their sensitivity to the quality of the training data and their high computational cost, more research is necessary.

Despite challenges, MRI‐only radiotherapy holds significant promise for the future of radiation therapy, potentially might improve treatment outcome for different cancer types, reduce radiation exposure, and enhance patient experience. sCT has the potential to be a valuable tool for PET attenuation correction in radiotherapy. With the advancement of technology, sCT applications in radiation therapy are expected to grow, providing more advanced and patient‐focused solutions for treatment delivery and planning.

### 5.5 Future directions

For sCT generation, there are several lines of research that remain to be explored. Among these directions are:

1. **Develop improved DL architectures**: More research work is needed for developing novel DL architectures that can produce more accurate and realistic sCT images. The potential of GANs for sCT generation can further be investigated particularly conditional GANs (cGANS), which can use MRI anatomical data to create sCTs that are more accurate and patient‐specific. Also, attention mechanisms can be incorporated within DL architectures so that the model may focus on important MRI anatomical features that have a major impact on CT image electron density. Furthermore, residual connections (ResNets) and densely connected networks (DenseNets) can also be explored to examine their effectiveness for capturing intricate relationships between CT attenuation values and MRI features.
2. **Enhance generalizability**: To improve generalizability, there is need to develop techniques for knowledge transfer between datasets to enhance the generalisation of DL models. Domain adaptation techniques can be exploited to adapt a model trained on one dataset to a different one, considering potential variations in MRI acquisition protocols or patient demographics. Also, unsupervised learning approaches can be utilized to pre‐train DL models on large, unlabeled MRI datasets to learn generalizable features applicable to different patient populations. Moreover, transfer learning with feature bottleneck layers can be explored as it aims to fine‐tune a pre‐trained model on a smaller, task‐specific dataset, using pre‐learned knowledge while adapting to specific requirements of sCT generation for a particular radiotherapy application.
3. **Incorporate past anatomy knowledge**: This study suggests the need of developing techniques for integrating past anatomy knowledge into the generation of sCTs. Past anatomical knowledge, such as historical CT scans or radiotherapy plans, can be incorporated into the sCT generation process to account for changes due to previous treatments. Additionally, integrating patient‐specific anatomical priors, such as age‐related variations or genetic information, could lead to more personalized and accurate sCTs.
4. **Improve robustness**: A bigger and more varied training cohort can be employed to increase the model's robustness to region‐specific problems. In this regard, data augmentation techniques like image rotation and noise addition can be implemented to increase the training dataset's size and diversity. Additionally, a method for identifying model‐specific flaws in quality assurance should be suggested before clinical implementation. In other words, the model can quantify its own uncertainty in the generated sCT, which can be used for quality assurance to identify potentially unreliable sCTs requiring further investigation by a medical professional.
5. **Preserve radiomic features**: New DL methods can be developed that can better preserve radiomic features in cross‐modal image synthesis tasks.^59^ Novel loss functions specifically tailored to sCT generation can be designed, prioritizing image similarity and radiomic features for treatment planning. Also, adversarial training frameworks can be explored where one network generates realistic sCTs and another discriminates real CTs from generated sCTs based on radiomic features, encouraging the sCT generation network to produce images that retain these features.
6. **Preserve data privacy in model's development through federated learning**: Considering the privacy concerns in oncology, where patient data privacy is of utmost importance, federated learning is a valuable approach for overcoming privacy concerns by enabling model training on decentralized data without revealing patient information.^88^ Another advantageous prospect of this recent ML technique is its capability to deal with distributed data resources. Access to a variety of patient databases is frequently restricted for oncology centers. Federated learning enhances model generalization by allowing institutions to work together without transferring data. Its substantial utilization has yet to be made in healthcare applications, including sCT generation for MRI‐only radiotherapy, having paramount potential for its efficacy. Techniques like secure aggregation or differential privacy ensure patient data security while enabling collaborative model training across institutions. On the other hand, federated learning platforms promote multi‐institutional collaboration, allowing institutions to leverage collective data resources without compromising patient privacy, accelerating model development, and improving generalizability.
7. **Standardization of validation processes**: There is more need of designing and implementing standardized techniques to assess the reliability and accuracy of sCT generation algorithms on various datasets and at various institutions.
8. **Multi‐modal learning**: To further increase the accuracy of sCT, there is significant potential of investigating the integration of additional data sources, such as functional MRI or PET scans.
9. **Clinical integration**: Further research, including well‐designed clinical trials, is required to thoroughly evaluate the impact of sCT‐based RTP on treatment efficacy and patient outcomes.

Exploring these future avenues in sCT generation using ML and DL to their full potential is necessary. Researchers may greatly increase the efficacy and efficiency of MRI‐only radiation planning by focusing on these areas in order to create more precise, reliable, and generalizable models.

## 6 CONCLUSIONS

This paper presents an overview of the advancements in sCT generation from MRI data for RTP; a rapidly evolving field that offers several advantages over traditional CT scans, such as reduced radiation exposure, improved treatment accuracy, and lower costs. This study reviewed various sCT generation techniques such as sequence‐based, Atlas‐based, segmentation‐based, DL‐based, and hybrid approaches which are among the most promising. sCT is used in clinical practice for MRI‐only treatment planning, adaptive radiotherapy, and PET/MR attenuation correction. Despite its potential benefits, it faces several challenges, including limited clinical experience, insufficient long‐term follow‐up data, image registration accuracy, tissue classification and segmentation, patient‐specific heterogeneity, integration into clinical workflows, and computational complexity. Clinical trials and real‐world applications are needed to validate the accuracy and reliability of sCT‐generated images. Accurate alignment between MRI and CT images is crucial for sCT generation, but challenges arise in maintaining alignment and addressing distortions. Customizing the process for each patient's unique characteristics is also essential for accurate treatment planning. Future research directions include developing more accurate and efficient sCT generation algorithms, standardizing validation procedures, and integrating sCT with other imaging modalities to provide a more comprehensive picture of the patient's anatomy and physiology.

## AUTHOR CONTRIBUTIONS

Mohamed A. Bahloul, Saima Jabeen, Sara Benoumhani, Zehor Belkhatir, and Areej Al‐Wabil performed research and analyzed data; Habib Abdulmohsen Alsaleh contributed to research discussions; Saima Jabeen and Sara Benoumhani wrote the initial version of the paper, all authors reviewed the paper.

## CONFLICT OF INTEREST STATEMENT

The authors declare no conflicts of interest.
